# The EPR effect and beyond: Strategies to improve tumor targeting and cancer nanomedicine treatment efficacy

**DOI:** 10.7150/thno.49577

**Published:** 2020-06-25

**Authors:** Yang Shi, Roy van der Meel, Xiaoyuan Chen, Twan Lammers

**Affiliations:** 1 Institute for Experimental Molecular Imaging, RWTH Aachen University Clinic, Aachen, Germany.; 2 Laboratory of Chemical Biology, Department of Biomedical Engineering and Institute for Complex Molecular Systems, Eindhoven University of Technology, Eindhoven, The Netherlands.; 3 Laboratory of Molecular Imaging and Nanomedicine, National Institute of Biomedical Imaging and Bioengineering, National Institutes of Health, Maryland, USA.; 4 Department of Targeted Therapeutics, University of Twente, Enschede, The Netherlands.; 5 Department of Pharmaceutics, Utrecht University, Utrecht, The Netherlands.

**Keywords:** EPR effect, enhanced permeability and retention (EPR), cancer nanomedicine, tumor targeting, active targeting, cancer immunotherapy, extracellular vesicles, imaging

## Abstract

Following its discovery more than 30 years ago, the enhanced permeability and retention (EPR) effect has become the guiding principle for cancer nanomedicine development. Over the years, the tumor-targeted drug delivery field has made significant progress, as evidenced by the approval of several nanomedicinal anticancer drugs. Recently, however, the existence and the extent of the EPR effect - particularly in patients - have become the focus of intense debate. This is partially due to the disbalance between the huge number of preclinical cancer nanomedicine papers and relatively small number of cancer nanomedicine drug products reaching the market. To move the field forward, we have to improve our understanding of the EPR effect, of its cancer type-specific pathophysiology, of nanomedicine interactions with the heterogeneous tumor microenvironment, of nanomedicine behavior in the body, and of translational aspects that specifically complicate nanomedicinal drug development. In this virtual special issue, 24 research articles and reviews discussing different aspects of the EPR effect and cancer nanomedicine are collected, together providing a comprehensive and complete overview of the current state-of-the-art and future directions in tumor-targeted drug delivery.

Formulating therapeutic molecules in nanocarriers to yield nanomedicines is an attractive approach to improve the therapeutic index of oncology drugs. Over the last three decades, the development of cancer nanomedicines has resulted in thousands of publications and several approved drug products for the treatment of solid and hematological malignancies. In the context of solid tumors, the **enhanced permeability and retention (EPR) effect** has become an important driver of cancer nanomedicine design and it has served as a key cornerstone of tumor-targeted drug delivery 1-3.

Recently, however, **the importance and the existence of the EPR effect** in human patients have been heavily debated 4-6. It has been demonstrated that the mechanism by which nanoparticles enter solid tumors is more complex than previously thought (potentially going beyond simple extravasation through gaps in the endothelial lining) 7, and that immune cells in the tumor microenvironment play important roles in nanomedicines' accumulation, retention and intratumoral distribution 8,9. In addition, it is clear that the EPR effect is significantly more pronounced in the small animal xenograft tumor models which are typically used to evaluate cancer nanomedicines in preclinical settings as compared to tumor growing in humans 10. Accumulation of nanocarriers in human tumors definitely does occur 11, but the extent varies heavily between patients and tumor types. Accordingly, quantifying the degree of EPR effect in tumors using non-invasive imaging is a promising approach to stratify patients for cancer nanomedicine treatment 12,13. Moreover, strategies are needed to improve the effectiveness of nanomedicine therapy. This can be done *via* pharmacological and physical co-treatments to prime tumors for improved delivery and efficacy, *via* active targeting, *via* the use of multi-stage and/or stimuli-responsive nanocarrier materials, and *via* the combination of nanotherapeutics with immunotherapy 14, which has already shown initial clinical success 15. In this virtual special issue of Theranostics, 24 research and review articles are compiled which discuss approaches aimed at improving the therapeutic efficacy of cancer nanomedicine. These strategies by themselves, and especially when combined with others, will improve cancer nanomedicine's clinical translation and ultimately improve patient outcomes 16-18.

Traditionally, EPR-mediated tumor accumulation is proposed to result from long-circulating nanoparticles with a hydrodynamic diameter size exceeding the renal clearance threshold, which can extravasate from leaky tumor vessels. However, recent studies have investigated approaches to **extend the conventional concept of EPR-based tumor targeting**. For example, Liu *et al.* describe the potential of exploring transcytosis for tumor targeting, which is a potential additional mechanism to mediate tumor targeting by nanomedicines, especially in highly stromal solid tumors such as pancreatic ductal adenocarcinoma with weak EPR effect 19. Bort and colleagues discuss studies on the use of ultrasmall nanoparticles for tumor targeting. These include polysiloxane-based nanoparticles with a hydrodynamic diameter of approximately 4 nm, which have been successfully tested in animal models and have recently entered a clinical trial for treating patients with brain metastases 20. In a comparative study, Xu *et al.* investigate the tumor targeting efficiency of ligand-modified nanoparticles of 3 and 30 nm, respectively. Their results show that functionalizing 3 nm nanoparticles with a targeting ligand increased tumor targeting efficiency and tumor penetration while this was not the case for 30 nm nanoparticles 21.

To improve the EPR effect and nanomedicine effectiveness, **pharmacological and physical co-treatments** have been employed to prime the tumor microenvironment. Kwon and colleagues summarize features of the tumor microenvironment that impair EPR-based tumor targeting by nanomedicines. In addition, several priming strategies to improve EPR effect are discussed, including physical and physiological measures to remodel the tumor microenvironment 22. Dhaliwal and Zheng focus on the applications of physical strategies to improve EPR effect of tumors including ultrasound and hyperthermia. The authors also summarize assessment methods and proper use of animal models to study EPR-mediated nanomedicine targeting 23. Among the physical strategies, Duan *et al*. discuss the applications of micro/nanobubbles to augment the thermal effect, acoustic streaming and cavitation mechanisms of ultrasound to enhance the EPR effect 24. Recognizing the importance of the vasculature in tumor development, Tsioumpekou *et al.* demonstrate that specific suppression of PDGFRβ kinase activity by 1-NaPP1 effectively modulates the tumor microenvironment by inhibiting angiogenesis 25.

**Active targeting** can be used as a complementary strategy to EPR-based passive targeting to improve nanomedicine tumor accumulation and retention. Tumor targeting ligands include antibodies, fragments of antibodies (e.g. nanobodies) and peptides. Dammes and Peer summarize the applications of monoclonal antibodies in molecular imaging of cancer, autoimmune disorders and cardiovascular diseases 26. In addition, the potential of using monoclonal antibody-based molecular imaging strategies in theranostics and precision medicine is highlighted. Oliveira and co-workers utilize epidermal growth factor receptor (EGFR)-targeted nanobodies to deliver photosensitizers to tumors for photodynamic therapy. Both monovalent nanobodies and biparatopic nanobodies are conjugated with photosensitizers. Although these two types of conjugates exhibit different biodistribution profiles, they result in similar levels of necrosis after photodynamic therapy, resulting in tumor reduction 27. Minko and colleagues report on the use of a synthetic luteinizing hormone-releasing hormone (LHRH) decapeptide for targeting lung cancer to deliver paclitaxel and siRNAs *via* nanostructured lipid nanoparticles. The nanomedicine was administered *via* inhalation which also showed efficient homing to target cells 28. Zhong and colleagues utilize cyclic RGD as a targeting ligand to improve the delivery of disulfide-crosslinked iodine-rich polymersomes to B16 melanoma. The actively targeted polymersomes exhibit an *in vivo* elimination half-life of 6.5 h in the blood circulation, thus achieving efficient tumor targeting (6.7 %ID/g) and displaying promising therapeutic efficacy 29.

Another strategy to improve cancer therapy is to employ **nanomedicine-based combination treatments**. Zhao *et al.* discuss the potential of this approach for the treatment of glioblastoma, benefiting from synergistic combinations of different therapeutic agents. The authors discuss the rationale of nanomedicine-based drug combinations and recent clinical progress in nanocarrier-based combination therapies 30. Yu *et al.* discuss a special class of nanomedicines which induce cancer starvation by anti-angiogenesis and vascular blockade 31. Such nano-interventions have been combined with other modalities such as chemotherapy, gene therapy and photodynamic therapy to achieve synergistic effects for cancer treatment. Zhu *et al.* report on a pH-sensitive nanomedicine formulation combining an enzyme, focused ultrasound-based tumor ablation and hypoxia alleviation to potentiate doxorubicin-based chemotherapy 32. Their catalase-loaded nanoparticles were able to increase oxygen levels in tumors by converting H_2_O_2_ to O_2_, which improved the effect of ultrasound ablation and reduced tumor hypoxia, and these effects together improved doxorubicin efficacy.

In addition to conventional nanocarriers used for EPR-based tumor targeting, new carriers based on **bio-inspired design and materials allowing for tumor-selective drug release** have been exploited. Wolfram and co-workers review the use of extracellular vesicle-based drug delivery systems 33. The intrinsic tissue tropism of extracellular vesicles is highly promising for tumor targeting and the authors summarize methods to load therapeutic agents in extracellular vesicle, and modification strategies to improve their tumor targeting ability. Mi summarizes nanomedicines with stimuli responsiveness for tumor targeted imaging, therapy and theranostics 34. Nanomedicines sensitive to endogenous and exogenous stimuli as well as their potential to improve therapeutic efficacy are discussed.

Cancer nanomedicines have been extensively combined with **immunotherapy** to improve treatment outcomes. Yu and colleagues summarize the recent progress of combination nano-immunotherapy, with a special focus on nanomedicines modulating the tumor immune microenvironment (TIME) to improve immunotherapeutic efficacy 35. An experimental report by Panagi *et al.* describes an immunomodulatory nanomedicine based on liposomes co-loaded with a transforming growth factor beta inhibitor and an immunogenic cell death inducer 36. The liposome-based combination treatment improves the immunogenicity of triple-negative breast tumors and potentiates the efficacy of checkpoint blockade antibodies.

**Imaging** is instrumental in tumor targeting and translational cancer nanomedicine, as it can help capture tumor targeting efficiency and the heterogeneity of the EPR effect in tumors. Miller, Weissleder and colleagues comprehensively review the advances in image-guided systems pharmacology of cancer nanomedicines 37. Recent developments of quantitative imaging technologies and their applications in systems pharmacology of nanomedicine are discussed, with a focus on utilizing computational modeling to understand and guide the manipulation of the EPR effect and tumor microenvironment for improving nanomedicine therapy. Dasgupta, Lammers *et al*. summarize the value of imaging-assistance in determining nanomedicine biodistribution, target site accumulation and drug release 38. Imaging techniques to eventually enable patient stratification *via* companion nanodiagnostics, *via* nanotheranostics, *via* conventional imaging techniques and *via* immunohistochemistry are discussed. A comprehensive review by De Maar, Deckers and colleagues addresses multiscale imaging techniques for analyzing the heterogeneity of nanomedicines' spatial distribution in tumors, which is an important - and often overlooked - reason for inefficient nanotherapy 39. The authors summarize the applications as well as the strengths and weaknesses of 3 classes of imaging techniques for assessing the intratumoral distribution of nanomedicines, *i.e.* non-invasive clinical imaging modalities (nuclear imaging, magnetic resonance imaging, computed tomography and ultrasound), optical imaging and mass spectrometry imaging. Moss and co-workers provide novel insight on the use of high-resolution *ex vivo* micro-computed tomography for studying the spatial distribution of liposomes in 4 different tumor models 40. Their work identifies vessel distribution and vessel support as crucial determinants of efficient liposome accumulation and distribution in tumors. Qi *et al.* report on the use of hyaluronic acid conjugated with fluorescent dyes for molecular imaging of pancreatic cancer in settings allowing for intraoperative imaging 41. Their results demonstrate that the molecular weight of hyaluronic acid and the physicochemical properties of conjugated dyes affect the efficiency of tumor-specific imaging. Finally, Goos *et al.* report on star polymers chelated with MRI contrast agents and radioisotopes for molecular imaging and endoradiotherapy of cancerous lesions *via* exploiting EPR-based tumor accumulation. In CT26 tumor-bearing mice, the star polymer-based nanoparticles demonstrated a very high tumor targeting efficiency (15-22 %ID/g), which contributed to the improved survival of mice upon endoradiotherapy intervention 42.

Altogether, this **Theranostics special issue** presents a timely and comprehensive collection of research and review articles focusing on the EPR effect and beyond. These articles summarize from various different angles our current understanding of nanomedicine-based tumor targeting, and they provide valuable expert perspectives on how to improve the use and the efficacy of (EPR-based) nanomedicine formulations for cancer therapy.
